# Supplementary material for: Non-Expressed Donor KIR3DL1 Alleles May Represent a Risk Factor for Relapse after T-Replete Haploidentical Hematopoietic Stem Cell Transplantation
Source: Cancers (Basel). 2023 May 13;15(10):2754. doi: 10.3390/cancers15102754 (PMC10216127; doi:10.3390/cancers15102754)
Supplement: Supplementary file 1 [file cancers-15-02754-s001.zip › cancers-2345444-supplementary.pdf]

								Expression on NK cell surface		
3DL1*	3DL1*	3DS1*		N	tel AA	tel AB	tel BB	3DL1	3DS1	
***004**	*001	-	-	24	24			High	-	
***004**	*002	-	-	12	12			High	-	
***004**	*015	-	-	11	11			High	-	
*001	*001	-	-	11	11			High	-	
*001	*002	-	-	9	9			High	-	
*002	*002	-	-	5	5			High	-	
*001	*008	-	-	4	4			High	-	
***004**	*008	-	-	3	3			High	-	
*002	*008	-	-	2	2			High	-	
*002	*015	-	-	2	2			High	-	
*002	*018	-	-	1	1			High	-	
*001	*015	-	-	1	1			High	-	
*001	*020	-	-	1	1			High	-	
*015	*015	-	-	1	1			High	-	
*008	*008	-	-	1	1			High	-	
*002	*020	-	-	1	1			High	-	
*020	*031	-	-	1	1			High	-	
*002	*005	-	-	8	8			High, Low	-	
*005	*015	-	-	5	5			Low, High	-	
*001	*007	-	-	4	4			High, Low	-	
*001	*005	-	-	4	4			High, Low	-	
*005	*008	-	-	3	3			Low, High	-	
*005	*020	-	-	2	2			Low, High	-	
*007	*008	-	-	2	2			Low, High	-	
*007	*020	-	-	1	1			Low, High	-	
*007	*015	-	-	1	1			Low, High	-	
*002	*007	-	-	1	1			High, Low	-	
***004**	*005	-	-	15	15			Low	-	
*005	*005	-	-	9	9			Low	-	
***004**	*007	-	-	4	4			Low	-	
*007	*007	-	-	2	2			Low	-	
*005	*007	-	-	2	2			Low	-	
***004**	*009	-	-	1	1			Low	-	
***004**	*069	-	-	1	1			Low	-	
***004**	***004**	-	-	10	10			**Null**	-	
***004**	***019**	-	-	1	1			**Null**	-	
***019**	***019**	-	-	1	1			**Null**	-	
*001		*013		10		10		High	+	
*002	*015	*013		7		7		High	+	2 alleles 3DL1, S1+
*002		*013		7		7		High	+	
*008		*013		5		5		High	+	
*015		*013		4		4		High	+	
*020		*013		3		3		High	+	
*001	*001	-	-	3		3		High	-	3DL1, 2DS4 2DS1+
*001		+		3		3		High	+	
*001		*049N		2		2		High	-	
***004**	*001	*013		1		1		High	+	2 alleles 3DL1, S1+
***004**	*002	*013		1		1		High	+	2 alleles 3DL1, S1+
*002		+		1		1		High	+	
*015	*015	-	-	1		1		High	-	3DL1, 2DS4 2DS1+
*015		*049N		1		1		High	-	
*002	*005	+		1		1		High, Low	+	2 alleles 3DL1, S1+
*005		*013		13		13		Low	+	
*005		+		3		3		Low	+	
*007		*013		3		3		Low	+	
***004**		*013		19		19		**Null**	+	
***004**		+		4		4		**Null**	+	
***019**		*013		2		2		**Null**	+	

***004**	***004**	-	-	2		2		**Null**	-	3DL1, 2DS4 2DS1+
***004**		*010		1		1		**Null**	+	
***019**	***019**	-	-	1		1		**Null**	-	3DL1, 2DS4 2DS1+
**-**	**-**	*013	*013	13			13	-	+	
				278	*167*	*98*	*13*			

Table S1 : KIR3DL1/S1 allele combinations and corresponding telomeric motifs and expression level on NK cell surface in 278 blood donors.

Telomeric (tel) motifs were defined taking into account KIR3DL1/3DS1/2DS1 and 2DS4 genes. TelAA individuals were characterized by the presence of KIR3DL1 and 2DS4 but the absence of KIR3DS1 and 2DS1 genes. TelAB individuals were characterized by the presence of KIR3DL1 and 2DS4 with 3DS1 and/or 2DS1. TelBB individuals were characterized by the presence of KIR3DS1 and/or 2DS1 but the absence of KIR3DL1 and 2DS4. KIR3DL1/S1^{+} NK-cell surface phenotype was determined from PBMC by 4-color multiparameter flow cytometry using the following mouse anti-human mAbs: anti-CD3-PerCP (SK7) and anti-CD56-allophycocyanin (B159) (BD Biosciences), anti-KIR3DL1/S1-PE (Z27 clone, Beckman Coulter, Marseille, France), and anti-KIR3DL1-FITC (DX9) (Beckman Coulter, Immunotech). Different levels of KIR3DL1 expression are observed on NK cell surface including Null, Low and High KIR3DL1 allotypes depending on KIR3DL1 alleles.

Characteristics	KIR3DL1 expressed<br>N = 144	KIR3DL1 null<br>N = 23	p-value[1]
age	58 (47, 65)	56 (49, 64)	>0.9
gender			0.3
female	59 (41%)	7 (30%)	
male	85 (59%)	16 (70%)	
disease			0.6
AML	69 (48%)	12 (52%)	
MDS	19 (13%)	3 (13%)	
MDS/MPN	4 (2.8%)	0 (0%)	
MPN	5 (3.5%)	0 (0%)	
IAA	3 (2.1%)	1 (4.3%)	
BPDCN	1 (0.7%)	0 (0%)	
B ALL	11 (7.6%)	1 (4.3%)	
T ALL	3 (2.1%)	1 (4.3%)	
HL	6 (4.2%)	3 (13%)	
NHL	12 (8.3%)	0 (0%)	
T NHL	8 (5.6%)	2 (8.7%)	
ProlymphoT	3 (2.1%)	0 (0%)	
DRI			0.2
low-inter	81 (60%)	10 (45%)	
high-very high	53 (40%)	12 (55%)	
Unknown	10	1	
status			0.2
CR1	59 (41%)	5 (23%)	
CR>1	18 (12%)	3 (14%)	
no CR	67 (47%)	14 (64%)	
Unknown	0	1	
conditioning			0.6
RIC	116 (81%)	17 (74%)	
MAC	2 (1.4%)	0 (0%)	
sequential	26 (18%)	6 (26%)	

Table S2. Characteristics of patients. Abbreviations: AML: acute myeloblastic leukemia; MDS: myelodysplastic syndrome; MPN: myeloproliferative neoplasm; MDS/MPN: mixed MDS/MPN syndrome; IAA : idiopathic acquired aplastic anemia; PBDCN: blastic plasmacytoid dendritic cell neoplasm; ALL : acute lymphoblastic leukemia; HL: Hodgkin lymphoma; NHL: non-Hodgkin lymphoma; prolymphoT: T prolymphocytic leukemia. DRI: disease risk index. CR1/>1: complete response n°1 or n°>1; RIC: reduced intensity conditioning; MAC: myeloablative conditioning. Continuous variables are given as median (interquartile range), and categorical variables as number (percentage). [1]Wilcoxon rank sum test; Pearson's Chi-squared test or Fisher's exact test when appropriate.

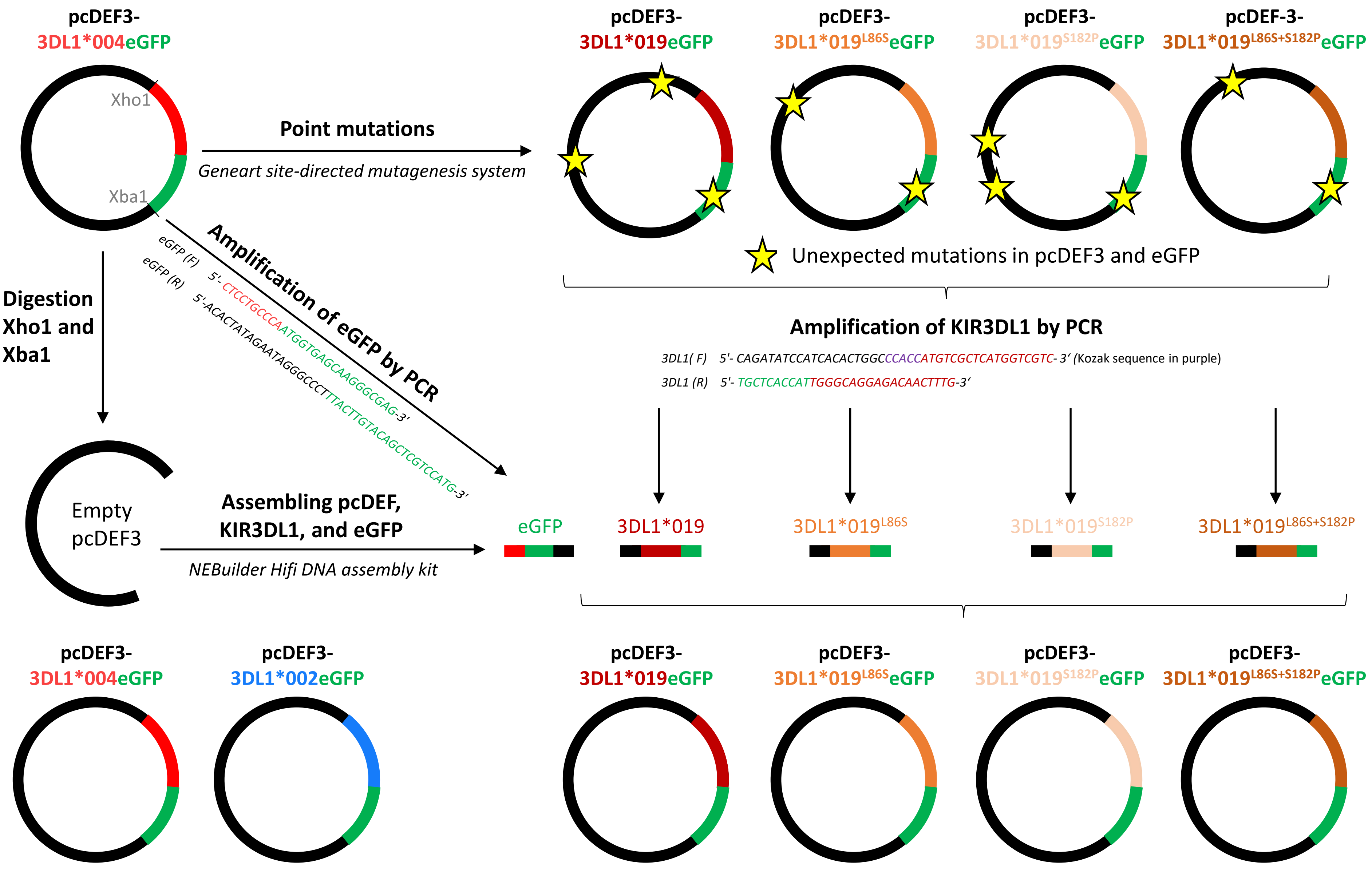

Figure S1: Representative illustration showing the strategy used to generate different KIR3DL1 constructs from the pcDEF3-3DL1*004-eGFP vector. Initial pcDEF3-3DL1*004 and pcDEF3-3DL1*002-eGFP are used as controls. Kits and corresponding primers used are shown in italics. KIR3DL1 and eGFP are shown using a specific color code.

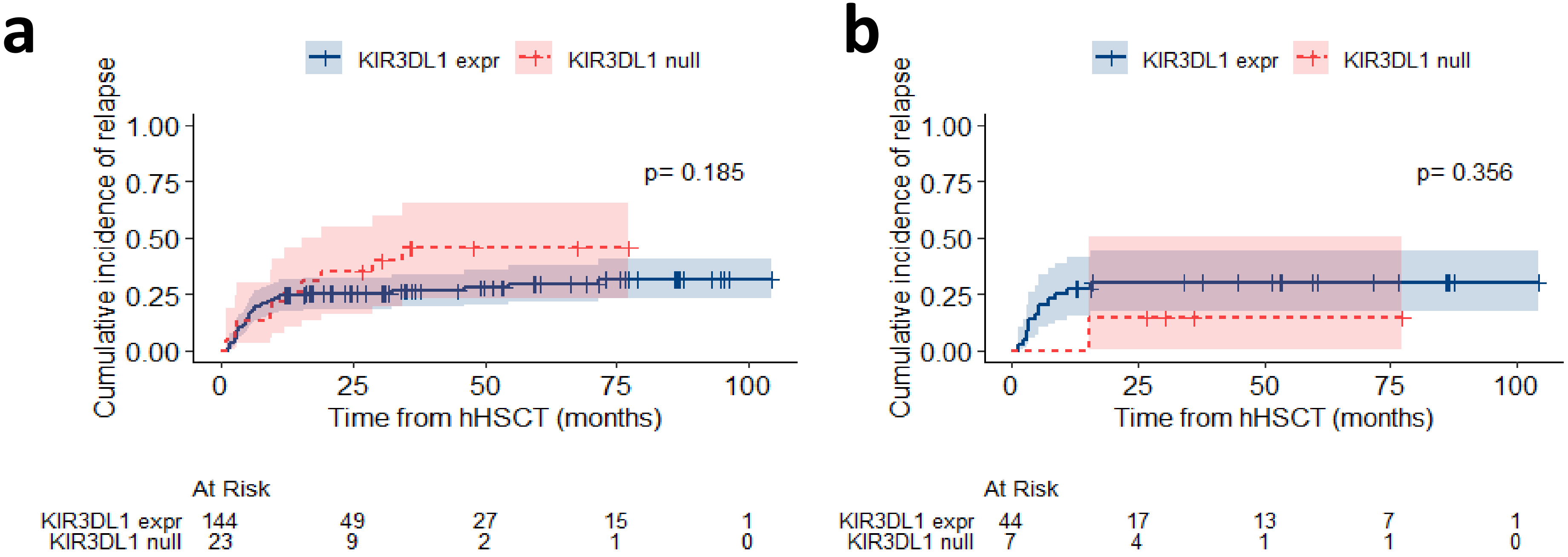

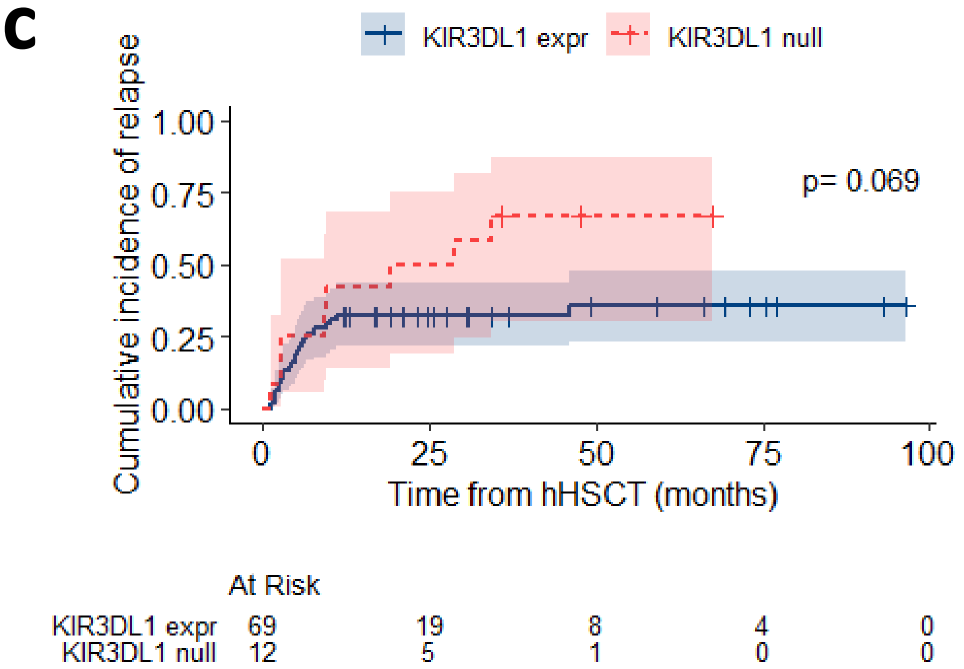

Figure S2 : Cumulative incidence of relapse after T replete haplo-identical HSCT (a) for all patients grafted with HSC donor non-expressed KIR3DL1 (n=23) or expressed KIR3DL1 (n=144), (b) for patients with lymphoid diseases grafted with HSC donor non-expressed KIR3DL1 (n=7) or expressed KIR3DL1 (n=44) and (c) for patients with acute myeloid leukemia grafted with HSC donor non-expressed KIR3DL1 (n=12) or expressed KIR3DL1 (n=69).
